# Histone deacetylase 3 (HDAC3) plays an important role in retinal ganglion cell death after acute optic nerve injury

**DOI:** 10.1186/1750-1326-9-39

**Published:** 2014-09-28

**Authors:** Heather M Schmitt, Heather R Pelzel, Cassandra L Schlamp, Robert W Nickells

**Affiliations:** Department of Ophthalmology and Visual Sciences, School of Medicine and Public Health, University of Wisconsin-Madison, 6640 MSC – 1300 University Ave, Madison, WI 53706 USA; Department of Biology, University of Wisconsin-Whitewater, Whitewater, WI 53190 USA

**Keywords:** HDAC, Epigenetics, Retinal ganglion cell, Neuronal degeneration, Apoptosis, Chromatin remodeling, Deacetylation, Heterochromatin

## Abstract

**Background:**

Optic nerve damage initiates a series of early atrophic events in retinal ganglion cells (RGCs) that precede the BAX-dependent committed step of the intrinsic apoptotic program. Nuclear atrophy, including global histone deacetylation, heterochromatin formation, shrinkage and collapse of nuclear structure, and the silencing of normal gene expression, comprise an important obstacle to overcome in therapeutic approaches to preserve neuronal function. Several studies have implicated histone deacetylases (HDACs) in the early stages of neuronal cell death, including RGCs. Importantly, these neurons exhibit nuclear translocation of HDAC3 shortly after optic nerve damage. Additionally, HDAC3 activity has been reported to be selectively toxic to neurons.

**Results:**

RGC-specific conditional knockout of *Hdac3* was achieved by transducing the RGCs of *Hdac3*^*fl/fl*^ mice with an adeno-associated virus serotype 2 carrying CRE recombinase and GFP (AAV2-Cre/GFP). Controls included similar viral transduction of *Rosa26*^*fl/fl*^ reporter mice. Optic nerve crush (ONC) was then performed on eyes. The ablation of *Hdac3* in RGCs resulted in significant amelioration of characteristics of ONC-induced nuclear atrophy such as H4 deacetylation, heterochromatin formation, and the loss of nuclear structure. RGC death was also significantly reduced. Interestingly, loss of *Hdac3* expression did not lead to protection against RGC-specific gene silencing after ONC, although this effect was achieved using the broad spectrum inhibitor, Trichostatin A.

**Conclusion:**

Although other HDACs may be responsible for gene expression changes in RGCs, our results indicate a critical role for HDAC3 in nuclear atrophy in RGC apoptosis following axonal injury. This study provides a framework for studying the roles of other prevalent retinal HDACs in neuronal death as a result of axonal injury.

**Electronic supplementary material:**

The online version of this article (doi:10.1186/1750-1326-9-39) contains supplementary material, which is available to authorized users.

## Background

Glaucoma is a leading cause of blindness worldwide and is characterized by damage to the optic nerve [1, 2]. Increased intraocular pressure results in an increase in the strain surrounding the optic nerve head [3, 4], which is believed to precipitate focal damage to retinal ganglion cell (RGC) axons as they pass into the optic nerve [5]. Axogenic neurodegeneration precedes somatogenic neurodegeneration in the predicted pathophysiology of a majority of optic neuropathies, such as glaucoma [6]. Optic nerve crush (ONC) mimics these molecular events by inducing partial RGC axonal damage [7], and it is a widely accepted model of acute RGC injury that has been used to study intrinsic and extrinsic apoptotic mechanisms of RGC death [7–12].

The intrinsic mechanism of apoptosis involves activation and translocation of the pro-apoptotic protein BAX to the mitochondria following cellular injury. Oligomerization of BAX at the mitochondrial outer membrane releases cytochrome c from the mitochondria [13], which leads to activation of caspases and subsequent cell death [14]. The steps involving BAX mark the committed step of intrinsic apoptosis [15]. In a previous study, *Bax-*deficient RGCs remained viable up to at least 72 weeks post ONC, however, these cells exhibited many early stages of atrophy typical of wild type cells undergoing cell death [8]. This was particularly evident in structural and functional changes in RGC nuclei. The RGC nuclei were found to exhibit atrophic characteristics including nuclear shrinkage, histone H4 deacetylation, heterochromatin formation, and RGC-specific gene silencing soon after ONC [8, 10, 16].

Changes in the transcriptional profile of damaged neurons have been described in several models of neurodegeneration, including the down regulation (silencing) of normal gene expression and the increase in expression of stress-response and pro-apoptotic genes [10, 17–26]. Transcriptional downregulation and initiation of the cell death mechanism in several cases of neuronal injury, including RGC death, were linked with epigenetic processes such as histone deacetylase (HDAC) activity [10, 18, 19, 27–29]. Although most HDACs are found ubiquitously in tissues, class I HDAC isoforms 1, 2, 3, and 6 are found primarily in the cells of the inner nuclear layer and the ganglion cell layer (GCL) of the murine retina [10, 30]. A previous study showed that RGC gene silencing and RGC death were attenuated following ONC as a result of pretreatment with the broad spectrum HDAC inhibitor Trichostatin A (TSA) [10]. The same study demonstrated that HDAC3 translocated to the nucleus in concert with H4 deacetylation during RGC death [10]. These results suggested a potential role for HDAC3 in early RGC gene downregulation and global deacetylation events in RGC death following axonal injury. Other studies have also reported that HDAC3 is toxic to differentiated neurons, indicating an important role for HDAC3 molecular events in neurodegeneration [28, 31, 32].

Here we show that conditional knock out of *Hdac3* in RGCs ameliorates global deacetylation and heterochromatin formation, while improving nuclear integrity and RGC viability following ONC. Interestingly, conditional knockout of *Hdac3* does not prevent the downregulation of RGC-specific gene expression, even though TSA does. We interpret these data as indicating that a different class I HDAC may be responsible for global transcriptional regulation in the early stage of nuclear atrophy. Overall, the results indicate an important role for HDAC3 in the early events of neuronal intrinsic apoptosis and provide direction for dissecting the roles of other class I HDACs in the process of early transcriptional silencing during the RGC apoptotic program.

## Results

### Intravitreal AAV2-Cre/GFP injection transduces ganglion cell layer of mouse retina

**Figure 1:**
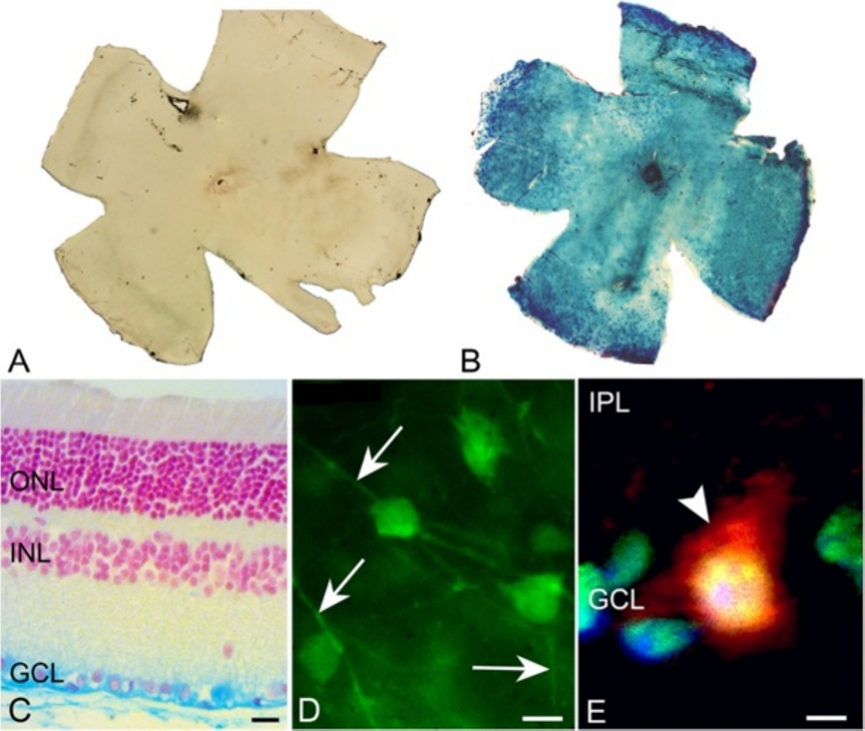
**AAV2-Cre/GFP transduces RGCs in** ***Rosa26-LacZ*** ^***fl/fl***^ **and** ***Rosa26-Tomato*** ^***fl/fl***^ **mice. (A-B)** Retinal whole mounts from non-injected **(A)** and injected **(B)** eyes of *Rosa26-LacZ* ^*fl/fl*^ mice were taken 8 weeks following injection. X-Gal staining indicates global reporter gene expression in injected eyes. **(C)** Retinal section taken from an injected eye of *Rosa26-LacZ* ^*fl/fl*^ illustrates that X-Gal staining is restricted to cells of the ganglion cell layer (GCL) (Scale bar: 20 μm) and is not present in the outer nuclear layer (ONL) and inner nuclear layer (INL). **(D)** GFP fluorescence carried by AAV2-Cre/GFP is found in the RGC somas and axons (indicated by arrows) in a retinal whole mount (Scale bar: 10 μm). **(E)** AAV2-Cre/GFP tropism to RGCs in the GCL, and not to the inner plexiform layer (IPL) is shown by nuclear BRN3A (green), TOMATO (red), and DAPI (blue) co-labeling in a retinal section (arrowhead). (Scale bar: 4 μm).

To selectively ablate *Hdac3* in RGCs, we transduced them with replication deficient AAV2 virus carrying a CRE expression cassette (AAV2-Cre/GFP). AAV2 has been reported to have selective tropism for RGCs [33]. To validate transduction of RGCs, we intravitreally injected C57BL/6-*Rosa26*^*fl/fl*^ mice containing either the *LacZ* or *Tomato* reporter gene and monitored reporter gene expression at times between 2 and 8 weeks of injection. In mice containing the *Tomato* reporter gene, fluorescence microscopy indicated that transduction of the ganglion cell layer plateaued by 4 weeks post intravitreal injection of AAV2-Cre/GFP (Additional file 1: Figure S1). It was also found that administration of 10^9^ genome copies of AAV2-Cre/GFP was sufficient to achieve maximal transduction in the retina. In mice containing the *LacZ* reporter, X-Gal staining revealed that reporter gene expression occurred in the ganglion cell layer (GCL) after intravitreal injection of AAV2-Cre/GFP (Figure 1A-C). We detected AAV2-Cre/GFP transduction of cells present in the GCL; predominantly, in BRN3A labeled RGCs as shown by fluorescent microscopy of injected *Rosa26-Tomato*^*fl/fl*^ mouse eyes (Figure 1D-E). The AAV2-Cre/GFP virus also transduced the occasional Müller cell (data not shown). No photoreceptors, or other neurons in the inner nuclear layer, were positively labeled for TOMATO or GFP.

### Knockdown of *Hdac3*expression after intravitreal injection of AAV2-Cre/GFP

**Figure 2:**
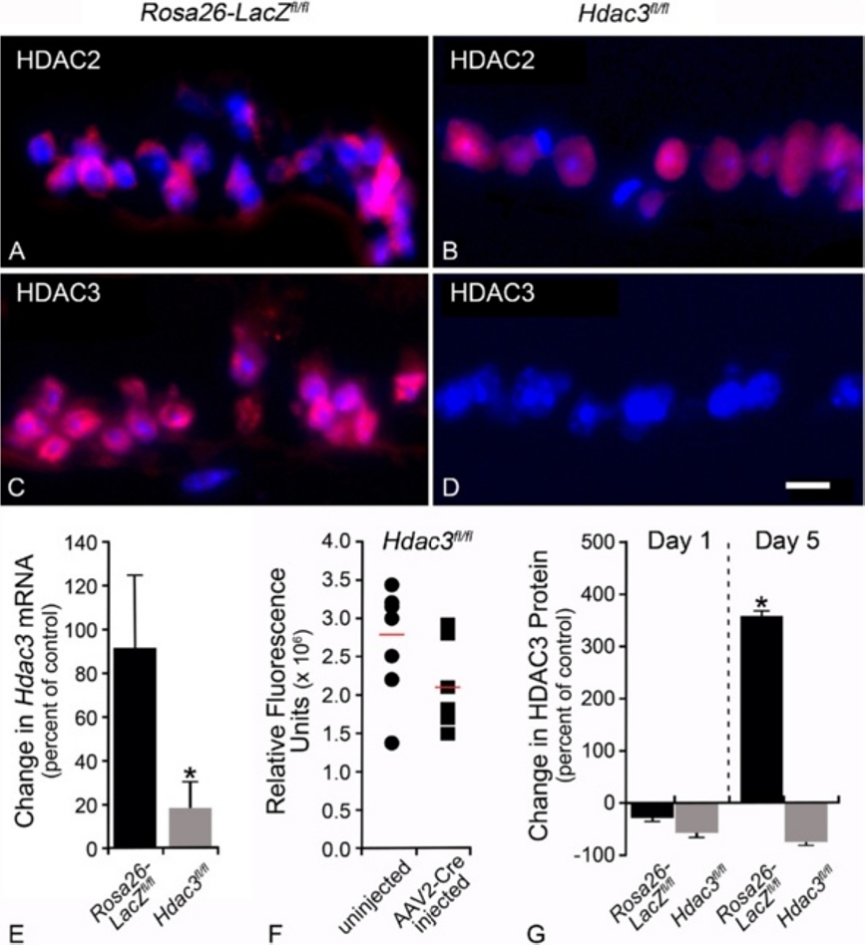
**Intravitreal AAV2-Cre/GFP injection achieved conditional knockout of the** ***Hdac3*** **gene. (A-B)** TEXAS RED labeling of HDAC2 in *Rosa26-LacZ* ^*fl/fl*^ and *Hdac3* ^*fl/fl*^ AAV2-Cre/GFP injected eyes at 5 days post ONC illustrates nuclear localization of this HDAC in cells of the GCL of both retinas. **(C-D)** TEXAS RED labeling of HDAC3 in *Rosa26-LacZ* ^*fl/fl*^ and *Hdac3* ^*fl/fl*^ AAV2-Cre/GFP injected eyes at 5 days post ONC demonstrates the lack of HDAC3 in the *Hdac3* ^*fl/fl*^ RGCs (Scale bar: 15 μm). **(E)** QPCR was used to determine abundance of *Hdac3* mRNA from pooled retinas of 5 *Rosa26-LacZ* ^*fl/fl*^ and *Hdac3* ^*fl/fl*^ mice at 4 weeks following AAV2-Cre/GFP injection. In uncrushed retinas, the abundance of *Hdac3* mRNA was significantly lower in *Hdac3* cKO retinas compared to *Rosa26-LacZ* ^*fl/fl*^ retinas (*P ≤ 0.05). **(F)** Protein from individual retinas of AAV2-Cre/GFP injected (4-week incubation) and uninjected *Hdac3* ^*fl/fl*^ mice showed an overall decrease in protein levels in *Hdac3* cKO mice, although there was no significant difference in mean abundance (P > 0.05). **(G)** Pooled retinal protein from 5 AAV2-Cre/GFP injected *Rosa26-LacZ* ^*fl/fl*^ and *Hdac3* ^*fl/fl*^ mice at 1 and 5 days post ONC were run on a Western blot. ONC results in an increase in HDAC3 accumulation in *Rosa26-LacZ* ^*fl/fl*^ mice by 5 days, but this response is significantly abrogated in *Hdac3* ^*fl/fl*^ animals (*P ≤ 0.05) when comparing *Rosa26-LacZ* ^*fl/fl*^ and *Hdac3* ^*fl/fl*^ mice at 5 days.

A previous study indicated that HDAC3 was recruited to the nuclei of RGCs following ONC, and that the mRNA abundance of both *Hdac2* and *Hdac3* increased at 1 and 3 days post ONC [10]. Here, we used fluorescence microscopy to monitor the expression of HDAC3 protein in cells of the GCL of *Hdac3*^*fl/fl*^ compared to *Rosa26-LacZ*^*fl/fl*^ mice, 5 days post ONC. Fluorescence microscopy images of retinal sections demonstrated that both *Rosa26-LacZ*^*fl/fl*^ and *Hdac3*^*fl/fl*^ mouse RGCs exhibited nuclear localization of HDAC2 (Figure 2A, B). Nuclear staining for HDAC3 was detected in the *Rosa26-LacZ*^*fl/fl*^ (Figure 2C), but absent in *Hdac3*^*fl/fl*^ mouse RGCs (Figure 2D). Quantitative measurements of the *Hdac3* mRNA also showed ~72% decrease in total retinal mRNA levels (Figure 2E, *P ≤ 0.05), while Western blotting for protein levels from individual retinas showed that 5/6 mice had reduced levels of HDAC3 in the injected eye (Figure 2F). Collectively, however, the overall levels of HDAC3 were not statistically significant. This discrepancy in mRNA and protein abundance may be due to the higher sensitivity of qPCR in comparison to Western blotting, or reflect differences in mRNA and protein stability in INL cells, which also express *Hdac3*. It was also found that HDAC3 protein levels increase ~350% by 5 days after ONC in *Rosa26-LacZ*^*fl/fl*^ mice, consistent with previous reports of increased amounts of *Hdac3* mRNA. Ablation of *Hdac3* in RGCs abrogates this damage-induced increase (Figure 2G, *P ≤ 0.05).

### *Hdac3*cKO leads to amelioration of global deacetylation following RGC injury

**Figure 3:**
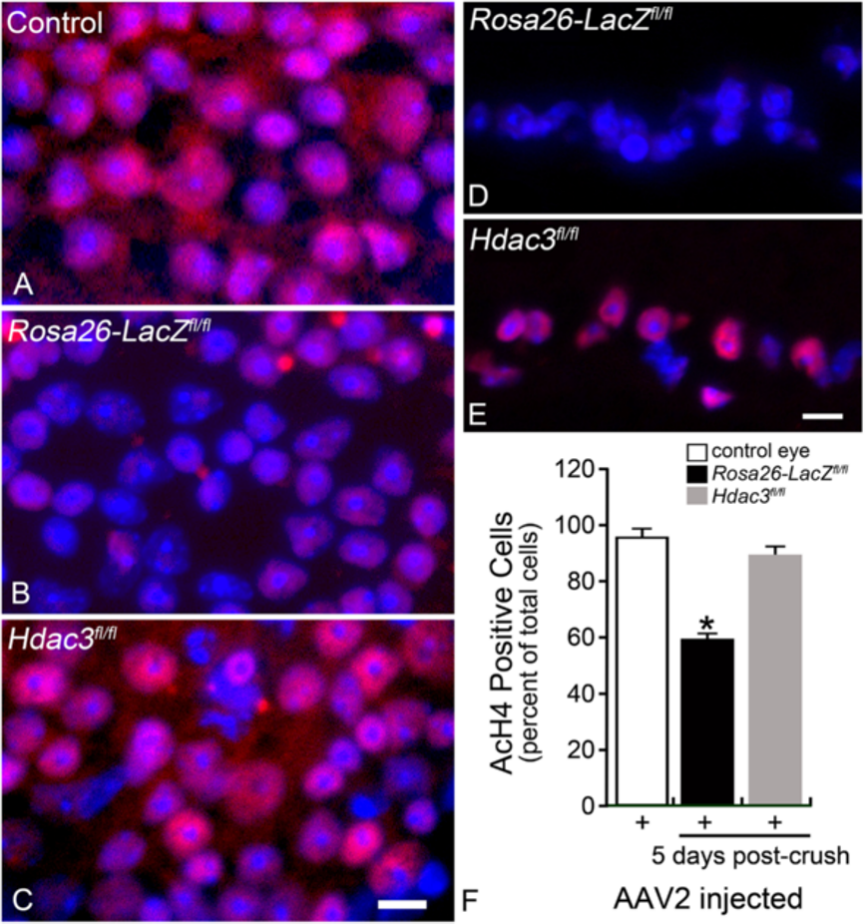
***Hdac3*** **cKO ameliorated global deacetylation following RGC injury. (A-C)** Retinal whole mounts were stained for AcH4 (red) in *Rosa26-LacZ* ^*fl/fl*^ and *Hdac3* ^*fl/fl*^ AAV2-Cre/GFP injected and uninjected eyes at 5 days following optic nerve crush. The control retina was an uninjected and uncrushed *Hdac3* ^*fl/fl*^ retina, which exhibited widespread AcH4 labeling (Scale bar: 10 μm). Nuclei absent of AcH4 staining were present only in the *Rosa26-LacZ* ^*fl/fl*^ retina after ONC. **(D-E)** Fluorescent microscopy of the retinal sections showed that the *Hdac3* cKO GCL retained visibly more AcH4 labeled cells than the Rosa26*-LacZ* ^*fl/fl*^ GCL (Scale bar: 15 μm). **(F)** Cell counts in the GCL indicated *Hdac3* cKO retinas at 5 days post ONC, retained AcH4 levels comparable to control retinas (P > 0.05), while *Rosa26-LacZ* ^*fl/fl*^ retinas exhibited about a 40% decrease in AcH4 labeled cells in the GCL compared to control retinas (*P ≤ 0.05).

Previously, it was shown that deacetylation of histone H4 occurred early in the apoptotic cascade initiated in RGCs as a result of ONC [8, 10]. Deacetylation was initiated within 24 hours following optic nerve damage, and peaked by 5 days. During the second and third days of this interval, HDAC3 was found localized to the nuclei of dying cells [10]. To investigate the specific role of HDAC3 in the process of H4 deacetylation, fluorescence microscopy was utilized to observe antibody staining of acetylated histone H4 (AcH4) in GCL nuclei at 5 days post ONC in *Hdac3*^*+/+*^*and Hdac3* cKO mouse eyes (Figure 3A-E). Results from GCL cell counts indicated that *Hdac3* cKO retinas retained comparable numbers of AcH4-positive nuclei in the GCL to that of untreated control retinas (P > 0.05). However, *Rosa26-LacZ*^*fl/f*l^ retinas that underwent ONC, exhibited a significant decrease of ~40% of positively labeled cells in the GCL (*P ≤ 0.05) (Figure 3F). This most likely represented loss of AcH4 in the nuclei of RGCs present in the GCL since RGCs make up ~50% of the population of neurons in this layer [34].

### *Hdac3*cKO blocks heterochromatin formation, nuclear envelope breakdown, and nuclear pore damage in RGCs following ONC

**Figure 4:**
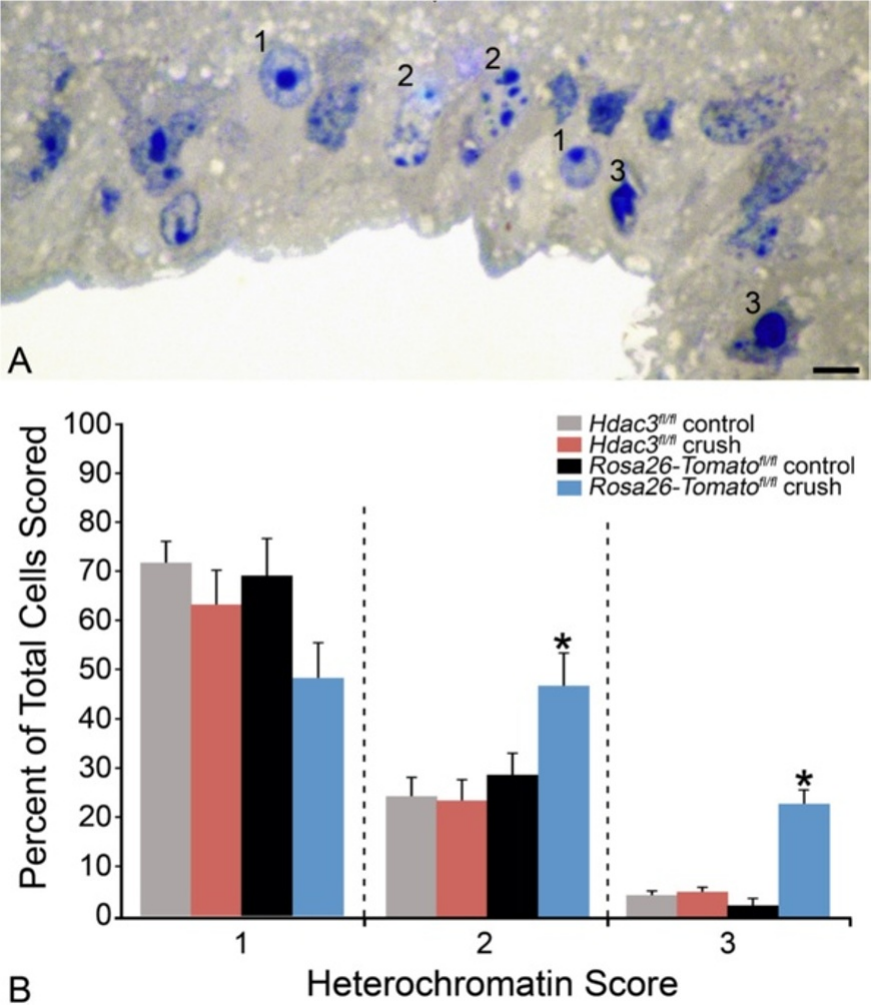
***Rosa26-Tomato*** ^***fl/fl***^ **retinas exhibited significantly more pyknotic nuclei at 5 days following ONC when compared to** ***Hdac3*** ^***fl/fl***^ **cKO and control retinas. (A)** A section of the retina from a *Rosa26-Tomato* ^*fl/fl*^ mouse, 5 days after ONC, to show an exemplar of the heterochromatin scoring system. **(B)** Scoring data from masked observers is depicted by a bar graph. Significantly more heterochromatic and pyknotic nuclei were detected in *Rosa26-Tomato* ^*fl/fl*^ control crushed retinas when compared to uncrushed and *Hdac3* cKO crushed retinas (*P < 0.05). Heterochromatin score of 1 = healthy cell with euchromatic nucleus and well-formed nucleolus, 2 = cell with partial apoptotic heterochromatin formation, and 3 = cell with completely heterochromatic (pyknotic) fragmented nuclei. (Scale bar: 10 μm).

**Figure 5:**
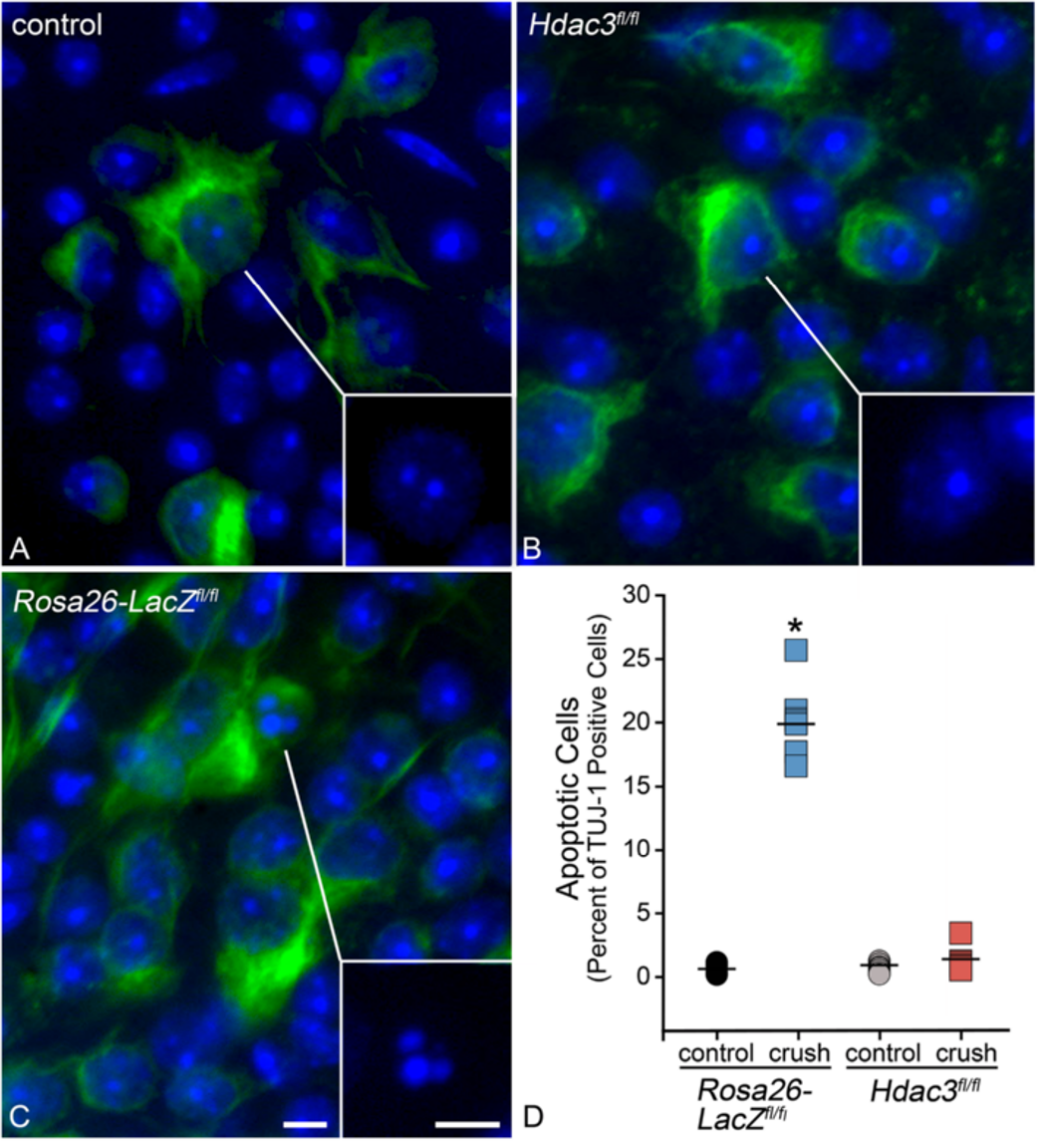
***Hdac3*** **cKO prevented apoptotic heterochromatin formation in TUJ-1 positive cells in the GCL 5 days post ONC. (A-C)** The GCL of whole mounts from a *Rosa26-LacZ* ^*fl/fl*^ control eye, a *Rosa26-LacZ* ^*fl/fl*^ crushed eye, and an *Hdac3* ^*fl/fl*^ crushed eye stained with the TUJ-1 monoclonal antibody. Nuclear morphology was stained using DAPI. The DAPI staining of a representative cells is shown in the inset in the lower right corner of each panel. Nuclei of TUJ-1 positive cells in control **(A)** and *Hdac3* ^*fl/fl*^ crushed retinas **(B)** typically exhibited a normal appearance with minimal heterochromatin and a prominent nucleolus. **(C)** In *Rosa26-LacZ* ^*fl/fl*^ retinas after ONC, however, some nuclei clearly showed condensed staining and were fragmented. **(D)** Graphical representation of cell counts indicating a significantly higher percentage of TUJ-1 positive apoptotic cells in *Rosa26-LacZ* ^*fl/fl*^ crushed eyes when compared to crushed *Hdac3* cKO and control eyes (*P ≤ 0.001). (Scale bars: 10 μm).

In addition to maximal histone deacetylation, 5 days after optic nerve crush also marks the point at which a significant number of affected cells exhibit the formation of heterochromatin [8]. To evaluate if HDAC3-mediated histone deacetylation correlates with heterochromatin formation at 5 days following ONC, cells in the GCL from *Rosa26-Tomato*^*fl/fl*^ and *Hdac3*^*fl/fl*^ retinal sections were scored on a scale of 1–3 based on images from bright field microscopy (Figure 4). Cells in the GCL from *Rosa26-Tomato*^*fl/fl*^ retinas scored significantly higher than in *Hdac3*^*fl/fl*^ retinas for fragmented and pyknotic nuclei (*P ≤ 0.05). To determine if the formation of pyknotic nuclei was restricted to RGCs, we also examined nuclear morphology of retinal whole mounts stained with DAPI and counter-stained with the TUJ-1 antibody, which has been reported to preferentially stain tubulin-rich RGCs [35]. Figure 5 shows that fragmented and pyknotic nuclei were significantly more numerous in *Rosa26-Tomato*^*fl/fl*^ mice after ONC (*P ≤ 0.05), while levels remained at control retina levels in *Hdac3*^*fl/fl*^ mice. Nuclear morphology in TUJ-1 negative cells in the GCL was not significantly affected by *Hdac3* cKO or ONC (data not shown).

**Figure 6:**
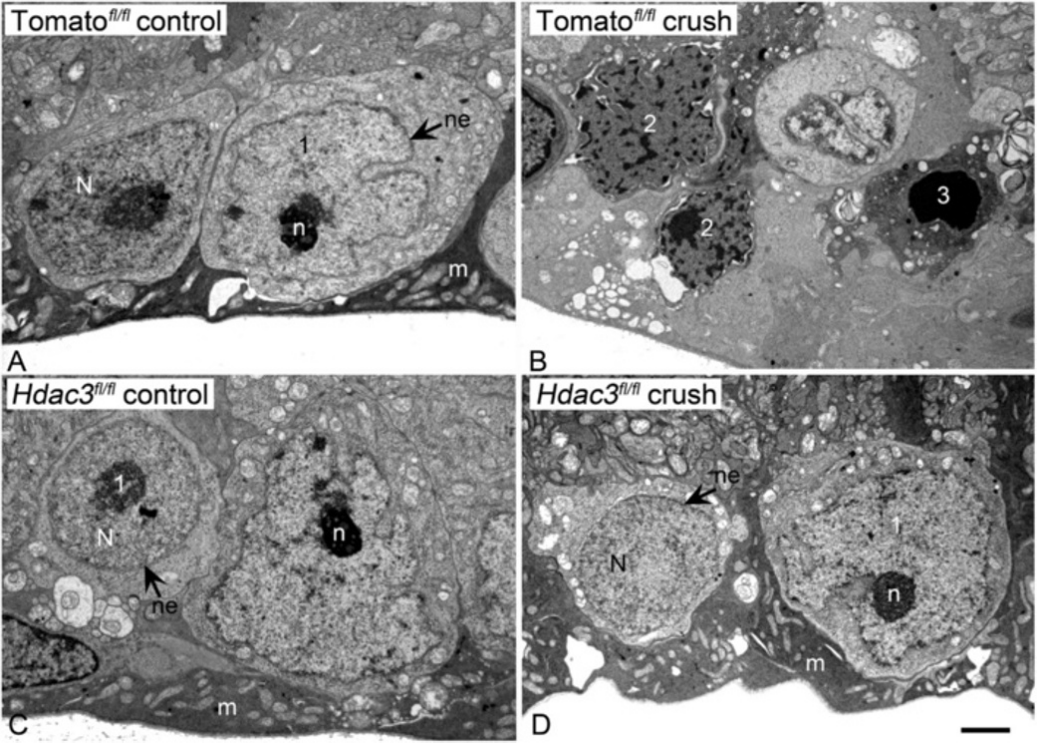
***Hdac3*** **cKO blocked heterochromatin formation in the GCL 5 days following ONC. (A-B)** TEM images of cells in the GCL of a *Rosa26-Tomato* ^*fl/f*l^ control eye and AAV2-Cre/GFP injected eye, 5 days after ONC. Prominent heterochromatin formation is evident in the GCL of *Rosa26-Tomato* ^*fl/f*l^ crushed retinas. **(C-D)** TEM images of cells in the GCL of an *Hdac3* ^*fl/fl*^ control eye and *Hdac3* cKO crushed eye indicate no heterochromatin formation in the GCL as a result of ONC. Nuclei with characteristics of the morphological scores described in Figure 4 are indicated. N = nucleus, n = nucleolus, ne = nuclear envelope, and m = Müller endfoot. (Scale bar: 3 μm).

Transmission electron microscopy (TEM) was also used to visualize heterochromatin formation in GCL cells 5 days post ONC in AAV2-Cre/GFP injected *Hdac3*^*fl/fl*^ and *Rosa26-Tomato*^*fl/fl*^ mice. TEM results demonstrated a higher incidence of early-apoptotic heterochromatic cells in the ONC *Rosa26-Tomato*^*fl/fl*^ retinas in comparison to the ONC *Hdac3*^*fl/fl*^ retinas (Figure 6A-D). These results suggest that HDAC3 plays an important role in heterochromatin formation in RGCs following axonal injury from ONC.

**Figure 7:**
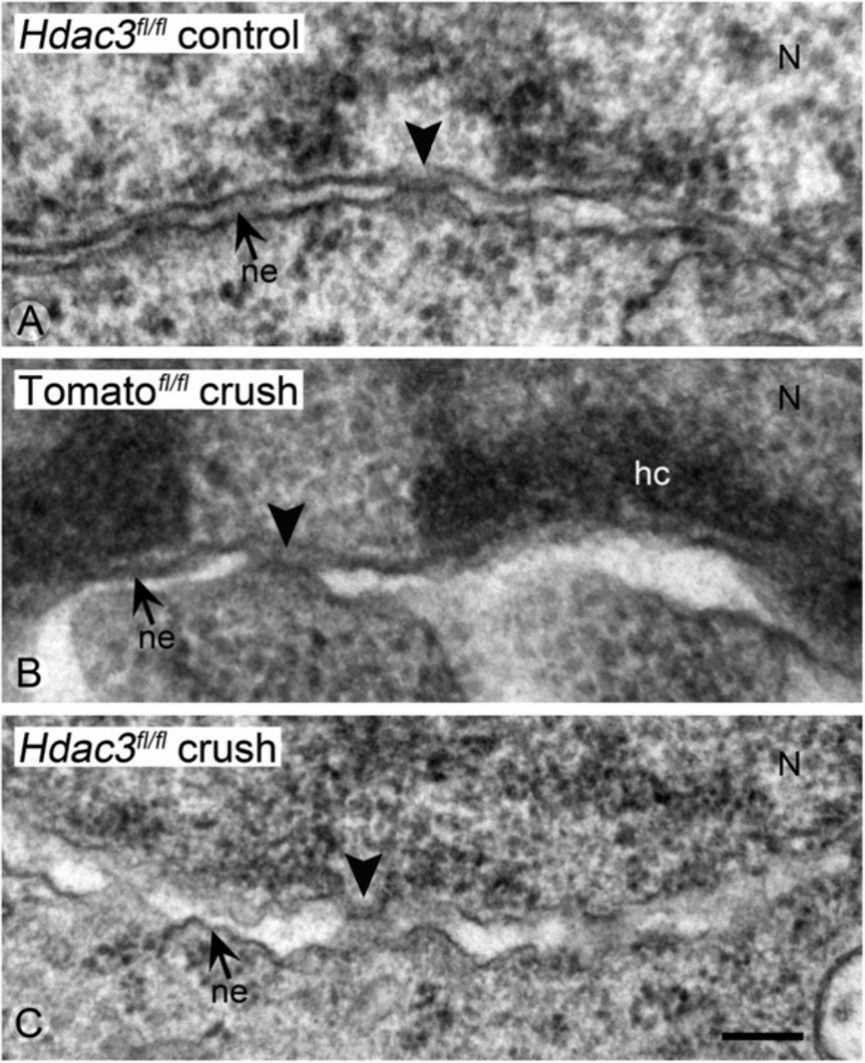
***Hdac3*** **cKO in the RGCs prevented the breakdown of the nuclear envelope and pore integrity following ONC. (A-C)** TEM images were taken of nuclear envelopes from *Hdac3* ^*fl/fl*^ control retinas, *Rosa26-Tomato* ^*fl/fl*^ crushed retinas, and *Hdac3* ^*fl/fl*^ crushed retinas. The nuclear lamina is situated at the top of each micrograph. **(A)** Example of the well-defined inner and outer membranes of the nuclear envelope and normal pore structure of a healthy cell. **(B)** Example of the nuclear envelope from a cell in a *Rosa26-Tomato* ^*fl/fl*^ retina 5 days after ONC. The nucleus exhibits formation of electron-dense heterochromatin localized to the inner surface of the nuclear envelope, and the intermembrane space has expanded. **(C)** The nuclear envelope of a presumptive RGC in an *Hdac3* ^*fl/fl*^ cKO mouse after ONC. The chromatin appears euchromatic and the nuclear envelop exhibits pore structures, although the apposition of the inner and outer membranes appears wavy. Arrowheads = nuclear pores, small arrows = nuclear envelope double membranes (ne), N = nucleus, and hc = heterochromatin. (Scale bar: 300 nm).

During the late stages of apoptosis, the nuclei of dying cells undergo nuclear envelope degradation and loss of nuclear pore integrity as a result of caspase-mediated lamin degradation. High magnification TEM imaging of nuclear envelopes revealed heterochromatin deposition adjacent to the inner surface of the inner membrane of cells in *Rosa26-Tomato*^*fl/fl*^ mice. The perinuclear space between the inner and outer membranes also appeared expanded (Figure 7B). Nuclear envelope structure in *Hdac3*^*fl/fl*^ mice exhibited minimal deposition of heterochromatin and normal appearing pore structures. The inner and outer membranes, however, often appeared separated and wavy (Figure 7C).

### Gene silencing is not affected by *Hdac3*cKO retinas in RGCs post ONC

**Figure 8:**
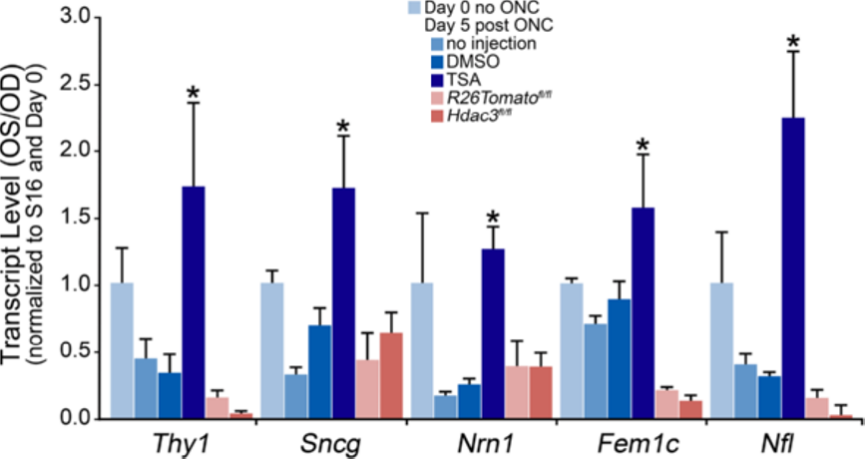
**Gene silencing was not regulated by HDAC3 in RGCs post ONC.** AAV2-Cre/GFP was injected intravitreally into *Rosa26-Tomato* ^*fl/fl*^ and *Hdac3* ^*fl/fl*^ eyes 4 weeks prior to ONC. Additional mice were injected intraperitoneally with either DMSO or TSA, 24 hours prior to ONC. Changes in gene expression for each group of treated mice were compared to mice that had received no injection of AAV2-Cre/GFP or HDAC inhibitor (no injection group). Transcript abundance of ganglion cell genes *Thy1, Sncg, Nrn1, Fem1c,* and *Nfl*, measured by qPCR, showed marked decreases in non-injected and DMSO injected as well as AAV2-Cre/GFP injected *Rosa26-Tomato* ^*fl/fl*^ and *Hdac3* ^*fl/fl*^ retinas at 5 days following ONC. No significant difference was observed among the change in transcript abundance between *Hdac3* cKO and the *Rosa26-Tomato* ^*fl/fl*^ mice in this study (P ≥ 0.05). However, mice injected intraperitoneally with TSA 24 hours prior to ONC exhibited significantly higher levels of mRNA abundance (*P ≤ 0.05) of RGC specific genes at 5 days following ONC when compared to uninjected and DMSO injected mice.

Previously, we found that the use of the broad-spectrum HDAC inhibitor, TSA, was effective in ameliorating RGC-specific *Fem1c* gene downregulation following ONC when administered intraperitoneally 24 hours prior to surgery [10]. To determine if HDAC3 activity regulates this response in dying RGCs, we quantified mRNA transcript levels in retinas from AAV2-Cre/GFP intravitreally injected *Rosa26-Tomato*^*fl/fl*^ and *Hdac3*^*fl/fl*^ mice at 5 days post ONC. RGC specific genes of interest included *Thy1*, *Sncg*, *Nrn1*, *Fem1c,* and *Nfl*. The abundance of these mRNAs was lower in the ONC retinas than in the unilateral uncrushed control eyes in both the AAV2-Cre/GFP injected *Rosa26-Tomato*^*fl/fl*^ and *Hdac3*^*fl/fl*^ mice (Figure 8). As a positive control, mRNA abundance was also quantified after pre-administration of TSA. Intraperitoneal injection of TSA, 24 hours prior to ONC, was able to attenuate the decrease in RGC-specific mRNA abundance when compared to uninjected and DMSO injected controls (*P ≤ 0.05). These results indicate that HDAC3 does not specifically regulate gene silencing following acute injury to RGCs, and they suggest that a different HDAC plays this role.

### RGC apoptotic death is attenuated in *Hdac3*cKO retinas post ONC

**Figure 9:**
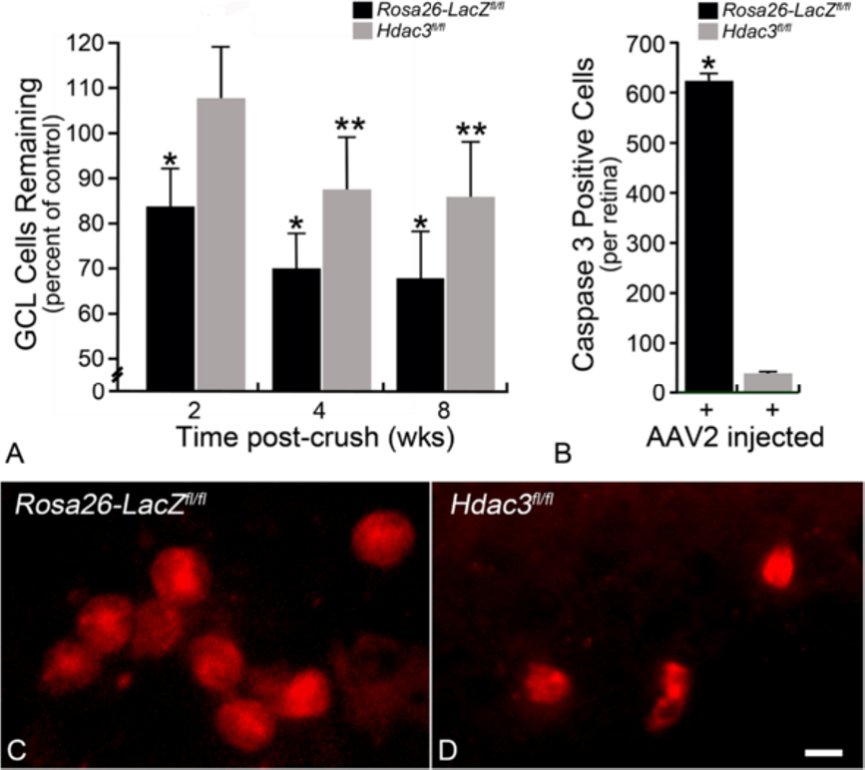
**RGC death was ameliorated in** ***Hdac3*** **cKO retinas at 2 weeks but not at 4 and 8 weeks following ONC. (A)** *Hdac3* ^*fl/fl*^ mice injected with AAV2-Cre/GFP retained a significantly higher percentage of the RGCs by 2, 4, and 8 weeks post ONC in comparison to injected *Rosa26-Tomato* ^*fl/fl*^ mice (*P ≤ 0.05). Retinas from *Hdac3* ^*fl/fl*^ mice injected with AAV2-Cre/GFP showed no significant cell loss at 2 weeks compared to contralateral eyes (P > 0.05), but did exhibit cell loss by 4 and 8 weeks post ONC (**P ≤ 0.05). **(B)** *Hdac3* ^*fl/fl*^ mice injected with AAV2-Cre/GFP also exhibited lower total numbers of CASPASE-3 positive cells 5 days following ONC in comparison to *Rosa26-LacZ* ^*fl/fl*^ injected and crushed retinas (*P ≤ 0.05). **(C-D)** Examples of CASPASE-3 positive cells in **(C)** *Rosa26-LacZ* ^*fl/fl*^ and **(D)** *Hdac3* ^*fl/fl*^ mice at 5 days post ONC. Images like these were used to collect the data graphed in **(B)**. (Scale bar: 10 μm).

Previous studies have shown that broad-spectrum HDAC inhibitors such as TSA and valproic acid (VPA) prevent RGC degeneration in models of retinal ischemic injury and optic nerve crush [10, 30, 36–38]. Others have shown that inhibition of HDAC1 and HDAC3 lead to RGC protection after neuronal injury [39]. HDAC3 has also been shown to be toxic to neurons in vitro [28, 39], and in models of degenerative disease such as Huntington’s disease, spinocerebellar ataxia type 7, and Friedrich’s ataxia [31, 40, 41]. Here, we assessed the effect of knockout of *Hdac3* on RGC cell survival after ONC. Cell counts in the GCL indicated that at 2 weeks following ONC, *Hdac3* cKO retinas had similar numbers (P > 0.05) of cells in the GCL when compared to its contralateral non-injected and uncrushed eye (Figure 9A). Conversely, injected *Rosa26-Tomato*^*fl/fl*^ retinas that underwent crush lost a significant percentage of cells from the ganglion cell layer (*P ≤ 0.05), which was comparable to the rate of cell loss in non-injected *Hdac3*^*fl/fl*^ and *Rosa26-Tomato*^*fl/fl*^ mice after ONC (data not shown). At 4 and 8 weeks post ONC, RGC numbers were significantly decreased (~20%) in the *Hdac3* cKO retinas in comparison to contralateral control retinas (**P ≤ 0.05), but this was still significantly less cell loss compared to *Rosa26-LacZ*^*fl/fl*^ retinas at all time points examined following ONC (*P ≤ 0.05) (Figure 9A). These results were similar to previous work that showed RGC protection by TSA treatment [16], indicating a critical role for HDACs, and especially HDAC3, in the process of RGC death.

Previous work has indicated that caspase activation occurs at 3–5 days following RGC axonal injury [42]. Therefore, to determine whether *Hdac3* cKO would ameliorate downstream apoptotic caspase activation, retinas from *Hdac3*^*fl/fl*^ and *Rosa26-Tomato*^*fl/fl*^ injected mouse eyes were stained with antibody to activated CASPASE-3 at 5 days post ONC. It was illustrated that retinas with cKO of *Hdac3* had significantly fewer positively labeled cells for activated CASPASE-3 in comparison to *Rosa26-Tomato*^*fl/fl*^ retinas (*P ≤ 0.05) (Figure 9B-D). These results suggest that early apoptotic events mediated by HDAC3 activity might be necessary for initiating the activation of downstream events in the apoptotic pathway.

## Discussion

The time course of RGC apoptosis can be temporally divided into phases of early cell and nuclear atrophy, initiation of BAX oligomerization, and late caspase and endonuclease activation leading to nuclear fragmentation. The main characteristics of the events of nuclear atrophy include gene silencing, global deacetylation, heterochromatin formation, and a decrease in nuclear structural integrity. Based on previous work showing neuronal toxicity of HDAC3, amelioration of RGC death with HDAC1 and HDAC3 inhibitors, and HDAC3 localization to the nuclei of RGCs prior to maximal histone H4 deacetylation during apoptosis [10, 28, 39], we sought to determine the role of HDAC3 in early RGC nuclear atrophy by analyzing each of the atrophic characteristics following ONC.

Here, we demonstrated that H4 deacetylation and heterochromatin formation were prevented in RGCs of *Hdac3* cKO retinas at 5 days following ONC. Although nucleoli and nuclear pores remained normal appearing, we found that the RGCs of *Hdac3* cKO retinas had wavy-appearing nuclear envelopes, which may reflect an intermediate stage of perinuclear swelling that was present in the nuclei of cells of crushed control mice. Alternatively, HDAC3 may play a role in maintaining the latticework that supports the inner nuclear membrane [43], by associating with lamin A/C (see below).

HDAC3 enzymatic activity, resulting in histone deacetylation and heterochromatin formation, are temporally situated early in the apoptotic program, because they still occur in *Bax-* deficient mice, where completion of apoptosis is effectively permanently blocked [8, 44]. It is not known, however, if these HDAC3-mediated changes are essential to activate further downstream events in the apoptotic pathway, such as caspase activation and nuclear fragmentation. Our results showing a correlation between HDAC3 activity and CASPASE-3 activation support a cause and effect relationship between these early events and later stages in the apoptotic pathway. Whether or not this relationship is linked to chromatin remodeling is not known at this time. HDAC3 may also play a role in modifying non-histone targets early in the apoptotic process. One such target may be the transcription factor p53. Acetylation and phosphorylation play a critical role in regulating p53 activity by altering cellular localization or binding affinity to specific targets [45]. Specifically, Chao and colleagues observed that HDACs could activate p53 by deacetylating lysine residue 317, leading to activation of pro-apoptotic gene expression such as *Bbc3 (PUMA)* and *Bim* [46] *.* Both of these BH3-only containing proteins of the *Bcl2* family of genes function by modulating the activity of BAX to promote mitochondrial permeability leading to the release of cytochrome-c and the activation of the caspase cascade. Importantly, both of these proteins have been strongly implicated in regulating RGC apoptosis after optic nerve damage [42]. Separately, Brochier et al. [47] observed that inhibition of p53 DNA-binding and transcriptional activity in neurons was obtained by acetylation on lysines 381 and 382. The same post-translational modifications enhance p53 activity in cancer cells, however. If HDAC3 plays such a role in regulating the acetylation status of p53, this would provide a mechanism for the selective toxicity of this HDAC in differentiated neurons [28, 40, 48].

One of the events in nuclear atrophy not affected by *Hdac3* deletion is the silencing of normal RGC gene expression. Previously, we showed that injection of broad-spectrum HDAC inhibitor, TSA, prior to ONC prevented silencing of the RGC specific gene promoter, *Fem1c,* in both acute and chronic (glaucomatous) models of axonal injury [10, 16]. We hypothesized that amelioration of gene silencing would occur with the conditional knockout of *Hdac3* in RGCs of mice that later underwent ONC. However, knockout of *Hdac3* did not lead to protection from gene silencing at 5 days following ONC, rather RGC specific gene transcript abundance also decreased similar to the change exhibited by wild type retinas. Conversely, TSA administration prior to ONC led to significant amelioration of RGC specific gene silencing in RGCs. These results, taken together with evidence that HDACs 1, 2, 3, and 6 localized in the retina [30], indicated a potential role for these other HDACs in the early event of gene silencing during RGC atrophy.

Several class I HDACs have been implicated in regulating gene transcription. An appropriate candidate for investigation may be HDAC2 due to it’s increased mRNA abundance in retinas at 1 day following ONC [10]. Previous work demonstrated that HDAC2 comprised approximately 35% of the total HDAC activity in the mouse retina and that retinas lacking HDAC2 underwent less retinal degeneration following ischemic insult [30]. The HDAC1/HDAC2 corepressor complex can be targeted to genes by transcription factors such as Sp1 and Sp3, playing a role in regulation of gene expression via chromatin remodeling [49].

A different mechanism of gene silencing, involving lamin-associated domains, was recently demonstrated in *Drosophila* S2 somatic cells, which exhibit silencing of a multi-genic testes gene cluster. This gene cluster is sequestered at the nuclear envelope as heterochromatin that interacts with the lamin protein complex. Domains, such as this one, are maintained by the activity of the *Drosophila* HDAC1 homolog, with the HDAC3 homolog playing an apparent auxiliary role [43]. Lamin-associated domains are increasingly recognized as a mechanism for repressing gene activity during development [50]. This phenomenon offers an interesting mechanism for gene silencing during cell death, and is consistent with one of the hallmarks of apoptosis, which is an initial accumulation of heterochromatin along the inner surface of the nuclear envelope [51, 52]. It is unknown if this accumulation of heterochromatin associates with lamin domains, or selectively involves aggregation of genomic DNA with actively transcribed genes that are targeted for silencing. This potential model warrants further investigation.

Although HDAC3, by itself, evidently does not seem to play a large role in early gene silencing, it may be a valuable molecular target for drug inhibitor therapy since knockout of *Hdac3* expression has been shown to halt the progression of histone deacetylation and attenuate subsequent RGC death after axonal injury. An important consideration resulting from these experiments is that HDAC-mediated changes in RGCs can be segregated into very early events, likely associated with gene-silencing, and later events, associated with global chromatin remodeling. It is still relevant to address the mechanism of gene silencing, since RGC function is expected to rely on these cells having a normal profile of gene expression that defines cell identity and function. We anticipate that selective targeting of HDAC3 for protective therapy may yield living, but non-functional RGCs, because of their inability to express a genetic profile that identifies them as ganglion cells. Further investigation into functions and timing of different HDAC activities during the process of chromatin remodeling will provide insight into early epigenetic events that play a role in RGC apoptosis associated with optic neuropathies.

## Conclusions

HDAC3 was found to play a major role in global deacetylation of histone H4, heterochromatin formation, and eventual cell death in RGCs that underwent axonal injury. However, deletion of HDAC3 did not affect gene silencing, even though the broad spectrum HDAC inhibitor was effective. This implies that other HDACs commonly found in the retina also play a role in early nuclear atrophic events during the intrinsic apoptotic program in RGCs.

## Methods

### Experimental animals, AAV2-Cre/GFP injection, and ONC

All mice were handled in accordance with the Association for Research in Vision and Ophthalmology statement for the use of animals for research, and experimental protocols were approved by the Institutional Animal Care and Use Committee of the University of Wisconsin. A random mixture of male and female C57BL/6 mice between the ages of 4–6 months, were used for experiments. The *Rosa26-LacZ*^*fl/fl*^ mice were generously provided by Dr. Jing Zhang from the University of Wisconsin-Madison McArdle Laboratory for Cancer Research (Madison, Wisconsin). *Rosa26-Tomato*^*fl/fl*^ mice were obtained from the Jackson Laboratory (Bar Harbor, Maine), and *Hdac3*^*fl/fl*^ mice [53] were obtained from Dr. Scott Hiebert at Vanderbilt University (Nashville, Tennessee, USA). The *Rosa26* mice contain the *loxP* flanked PGK-Neo stop transcript found upstream of either *Tomato* or *LacZ* (βGEO) reporter transcript and were used to monitor for RGC transduction of the AAV2-Cre/GFP virus as well as *Hdac3*^+/+^ controls for viral injections (see below). Each treatment group contained at least 4 mice for all experiments except for heterochromatin formation analysis (n = 3). The C57BL/6 *Hdac3*^*fl/fl*^ mice contain loxP sites flanking exon 7 of the *Hdac3* gene transcript [53].

Injections of 1 μL AAV2-Cre/GFP (equaling a total of 10^9^ gc) were administered intravitreally 4 weeks prior to ONC. The 4-week time point was chosen after testing transduction efficiency at 1, 2, 4, and 8 weeks post AAV2-Cre/GFP injection showed optimal expression of *Tomato* starting at 4 weeks (Additional file 1: Figure S1). AAV2-Cre/GFP was obtained from Vector Biolabs (Philadelphia, PA, USA), and 1:10 dilution of stock AAV2-Cre/GFP was made in 5% glycerol in sterile phosphate buffered saline (PBS, 137 mM NaCl, 1.8 mM KH_2_PO_4_, and 10 mM Na_2_HPO_4_, pH 7.5) prior to injection. Intravitreal injections were conducted using a 10 μL Hamilton syringe with a 35G needle attached. A volume of 1 μL of diluted AAV2-Cre/GFP was injected over a period of 40 seconds, and the needle was held in the eye for at least 30 seconds before it was retracted. ONC was performed unilaterally using self-closing forceps to initiate degeneration of RGCs. Retinas were harvested at 5, 14, 28, and 56 days following ONC for analysis. Previously, we observed peak histone H4 deacetylation in the GCL by 5 days post-ONC [10]. Therefore, retinas were harvested at 5 days to assess mRNA abundance, histone H4 deacetylation, and heterochromatin formation. Retinas were collected at 14, 28, and 56 days post ONC for cell counts. Cell counts were obtained using a modification of the method described in Li et al. (2007) [54]. Briefly, digital images collected at 400× magnification were taken of each lobe of a retinal whole mount stained with DAPI. Cell numbers were determined in 24 separate 100 μm^2^ fields for each retina. Change in cell number for each experimental eye was calculated as a percentage of cell numbers in the corresponding control eye of each mouse.

### β-galactosidase staining and bright field microscopy

*β-galactosidase* reporter expression was identified histochemically in retinal sections and whole mounts by X-Gal assay. X-gal staining solution was prepared ahead of time in the dark by adding N, N dimethylformamide to X-gal (0.02% Igepal, 0.01% sodium deoxycholate, 5 mM potassium ferricyanide, 5 mM potassium ferrocyanide, and 2 mM MgCl_2_ diluted in 0.1 M PBS [pH 7.3]). Mice were euthanized and eyes were collected and fixed in 4% paraformaldehyde in PBS at room temperature for 50 minutes. For whole mounts, the eye was then rinsed with PBS, and the anterior portion of the eye was removed, leaving an eyecup. The eyecup was then washed in PBS containing 2 mM MgCl_2_ and 2 μM CaCl_2_ and stained by incubation in staining solution containing 1 mg/mL X-gal at 37°C for 18 hours. After staining, the retina was dissected from the eyecup and whole mounted on glass slides. For sections, the eye was rinsed with PBS, and the anterior portion of the eye was removed, leaving an eyecup. The eyecup was then equilibrated in 30% sucrose overnight at 4°C before mounting in blocks of Tissue-Tek O.C.T. Compound from Fisher Scientific (Pittsburgh, PA) for cryosectioning (5 μm thick). Sections were then rinsed in PBS containing 2 mM MgCl_2_ and 2 μM CaCl_2_ and stained by incubation in staining solution containing 1 mg/mL X-gal at 37°C for 18 hours. Retinas were then washed in PBS and stained with nuclear fast red stain for 5 minutes. The slides were examined and photographed using an Olympus BX40 light microscope (Olympus America Inc., Center Valley, PA) and digital camera attachment.

### Immunofluorescence

Indirect immunofluorescence on 5 μm thick frozen retinal sections and whole mounts was done as described previously [10]. Cryosections and whole mounts were mounted on Superfrost Plus microscope slides (Fisher Scientific) and rinsed in PBS. The sections were then blocked in 5% bovine serum albumin (BSA) in PBS for 3 hours at room temperature and later rinsed in PBS. Primary antibodies including polyclonal rabbit antibody to human HDAC3 (#sc-11417) and polyclonal rabbit antibody HDAC2 (#sc-7899) (both from Santa Cruz, Dallas, TX), polyclonal rabbit antibody to human AcH4 (#06-866) and monoclonal mouse primary to human BRN3A (#MAB1585) (both from EMD Millipore Inc., Billerica, MA), monoclonal mouse primary to human TUJ-1 (#ab14545) (AbCam, Cambridge, MA), and polyclonal rabbit primary antibody to human CASPASE-3 (#AF835) (R&D Systems, Minneapolis, MN) were used at 1:100 dilutions. Sections and whole mounts were incubated in primary antibody for 24–48 hours at 4°C and washed in PBS afterwards. Secondary antibodies used included goat anti-rabbit TEXAS RED (1:1,000) and goat anti-mouse FITC (1:1,000) (Jackson ImmunoResearch Laboratories, West Grove, PA). Sections and whole mounts were incubated in secondary antibody at room temperature in the dark for 2 hours and washed in PBS. All sections and whole mounts were counter-stained for 10 minutes with 4’, 6-diamidino-2-phenylindole (DAPI) and were washed in PBS. Finally, sections and whole mounts were mounted using Immumount mounting medium (Fisher Scientific) and coverslipped. Fluorescent images were obtained using a Zeiss Axioplan 2 Imaging microscope with Axiovision 4.6.3.0 software (Carl Zeiss MicroImaging Inc., Thornwood, NY).

### Transmission Electron Microscopy (TEM)

*Rosa26-Tomato*^*fl/fl*^ and *Hdac3*^*fl/fl*^ mouse eyes were injected with AAV2-Cre/GFP and after 4 weeks were subjected to ONC surgery. Animals were analyzed 5 days after ONC surgery. Enucleated eyes were immersed in 4% paraformaldehyde in 0.1 M Phosphate buffer (PB) for 5 minutes, after which the anterior chambers and lenses were dissected away from each eyecup. A small region of the superior eyecup was then removed and placed in 2.5% glutaraldehyde, 2% paraformaldehyde in PB overnight at 4°C. Tissues were postfixed in 1% osmium tetroxide in PB, dehydrated in ethanol, and embedded in Epon epoxy. Sections (60–90 nm) were cut, stained with 50% ethanoic uranyl acetate and Reynold’s lead citrate, and viewed using a Phillips CM120 transmission electron microscope (FEI Company, Hillsboro, OR).

### Heterochromatin scoring analysis

Tissues processed for TEM were also sectioned for bright field microscopy. Thick (1 μm) sections were cut from epoxy embedded samples and stained with Richardson’s stain (methylene blue and azure blue). Sections were imaged using an Olympus BX40 light microscope and a digital camera attachment. Nuclear morphology of cells in the GCL was scored by 3 masked observers. A score of 1 indicated cells that exhibited healthy euchromatic nuclei with well-formed nucleoli; a score of 2 indicated cells that were partially heterochromatic; and a score of 3 indicated cells that had completely condensed pyknotic chromatin or fragmented nuclei.

### Evaluation of transcript abundance in the retina by qPCR

Total retinal RNA was isolated from 5 pooled retinas at 5 days post ONC by acid-phenol extraction, and RNA was then DNase I treated (Promega, Madison, WI). First strand cDNA using reverse transcriptase and oligo (dT) was synthesized from 2 μg of isolated and purified total RNA [55]. The resulting cDNA was diluted 10-fold and 5 μl of cDNA was used for each qPCR reaction with SYBR Green PCR master mix (Applied Biosystems, Foster City, CA) and the appropriate RGC gene-specific primers as listed in the table of primer sequences (Table 1). Quantitative PCR was conducted on triplicate samples in each run using ABI 7300 Real Time PCR system (Applied Biosystems). Data were obtained from triplicate samples for each target cDNA. Absolute transcript quantification was based on a standard *S16* curve run during the same reaction and the copy number was normalized to *S16* ribosomal protein mRNA. The mRNA transcript values are expressed as the percent change from contralateral control eye to treatment eye. Data were reported as the mean ± SD of these differences.

**Table 1:** **Primers for qPCR analysis**

Gene name	Primer sequences	Size of product (bp)
*Thy1*	5’-CTTGCAGGTGTCCCGAGGGC-3’	379
	5’-CTGAACCAGCAGGCTTATGC-3’	
*Sncg*	5’-GACCAAGCAGGGAGTAACGG-3’	240
	5’-TCCAAGTCCTCCTTGCGCAC-3’	
*Nrn1*	5’-TTCACTGATCCTCGCGGTGC-3’	238
	5’-TACTTTCGCCCCTTCCTGGC-3’	
*Nfl*	5’-AGCACGAAGAGCGAGATGGC-3’	173
	5’-TGCGAGCTCTGAGAGTAGCC-3’	
*S16*	5’-CACTGCAAACGGGGAAATGG-3’	198
	5’-TGAGATGGACTGTCGGATGG-3’	

All primers were designed to span at least one intron. For each gene, the forward primer is shown first.

### Western blot analysis

Western blot analysis was conducted on 5 pooled *Tomato*^*fl/fl*^ and *Hdac3*^*fl/fl*^ mouse retinas from each treatment group described. Individual retinas harvested from *Hdac3* cKO and control eyes were also analyzed. Retinal protein was loaded in triplicate with 50 μg per lane on 12% polyacrylamide gels and transblotted onto Immobilon P (Millipore, Inc., Billerica, MA). Membranes were probed for HDAC3, HDAC2, and ACTIN. Rabbit polyclonal antibodies were used at 1:1,000 for HDAC3 and HDAC2 and a goat monoclonal antibody was used at 1:250 for ACTIN (I-19) (cat# sc-1616) (Santa Cruz, CA). Firstly, the blots were incubated in donkey anti-goat secondary (1:10,000) conjugated to IRDye 800CW (cat# 926–32214), and after washing in PBS, incubated in goat anti-rabbit secondary (1:10,000) conjugated to IRDye 680RD (cat# 926–68071) (LICOR, Lincoln, NE). Images were scanned and analyzed using the Odyssey Clx (LICOR). Band fluorescence was quantified using Image Studio software, and data were normalized to the ACTIN loading control on each blot.

### Statistical analysis

Data were collected from a minimum of 4 independent samples in all experiments except for analysis of heterochromatin formation (n = 3), and shown as the mean ± standard deviation in all experiments except for cell counts, where data was shown as the mean ± standard error. All statistical analyses were performed using either the Student’s *t*-test with statistical significance set at P ≤ 0.05 for comparison of two groups or ANOVA with Bonferroni adjustments with statistical significance set at P ≤ 0.05 for comparison of multiple groups.

## Electronic supplementary material

Additional file 1: Figure S1: Optimal viral transduction and gene expression following intravitreal injection of AAV2-Cre/GFP. Outer panels indicate high magnification images of cells from the ganglion cell layer of retinal whole mounts from *Rosa26-Tomato* ^*fl/fl*^ mice intravitreally injected with AAV2-Cre/GFP 2 and 8 weeks prior. (Scale bar: 10 μm) The inner panels illustrate low magnification images of whole-mounted retinas from the same *Rosa26- Tomato* ^*fl/fl*^ mice that were intravitreally injected 1, 2, 4, and 8 weeks prior to imaging. Expression of *td-Tomato* peaks as early as 4 weeks post AAV2-Cre injection. (PDF 6 MB)

## Abbreviations

ONC: Optic nerve crush
RGC: Retinal ganglion cell
GCL: Ganglion cell layer
INL: Inner nuclear layer
IPL: Inner plexiform layer
ONL: Outer nuclear layer
HDAC: Histone deacetylase
AcH4: Acetylated histone 4
TEM: Transmittance electron microscopy
βGeo: β-Galactosidase and neomycin phosphotransferase fusion reporter protein
X-gal: (BCIG) for 5-bromo-4-chloro-3-indolyl-β-D-galactopyranoside
TSA: Trichostatin A
VPA: Valproic acid
PBS: Phosphate-buffered saline
BSA: Bovine serum albumin
DMSO: Dimethyl sulfoxide
DAPI: 4’, 6-diamidino-2-phenylindole
cKO: Conditional knockout
AAV2-Cre/GFP: Adeno-associated virus serotype 2 carrying Cre recombinase and green fluorescent protein
PGK-neo: Phosphoglycerate kinase I neomycin resistance gene
gc: Genome copies
N: Nucleus
n: Nucleolus
ne: Nuclear envelope
m: Müller endfoot
hc: Heterochromatin
WT: Wild-type.
