# Supplementary material for: Histone deacetylase 3 (HDAC3) plays an important role in retinal ganglion cell death after acute optic nerve injury
Source: Mol Neurodegener. 2014 Sep 28;9:39. doi: 10.1186/1750-1326-9-39 (PMC4190472; doi:10.1186/1750-1326-9-39)
Supplement: Supplementary file 1 — Additional file 1: Figure S1: Optimal viral transduction and gene expression following intravitreal injection of AAV2-Cre/GFP. Outer panels indicate high magnification images of cells from the ganglion cell layer of retinal whole mounts from Rosa26-Tomato fl/fl mice intravitreally injected with AAV2-Cre/GFP 2 and 8 weeks prior. (Scale bar: 10 μm) The inner panels illustrate low magnification images of whole-mounted retinas from the same Rosa26- Tomato fl/fl mice that were intravitreally injected 1, 2, 4, and 8 weeks prior to imaging. Expression of td-Tomato peaks as early as 4 weeks post AAV2-Cre injection. (PDF 6 MB) [file 13024_2014_549_MOESM1_ESM.pdf]

Additional File 1

Supplemental Figure 1

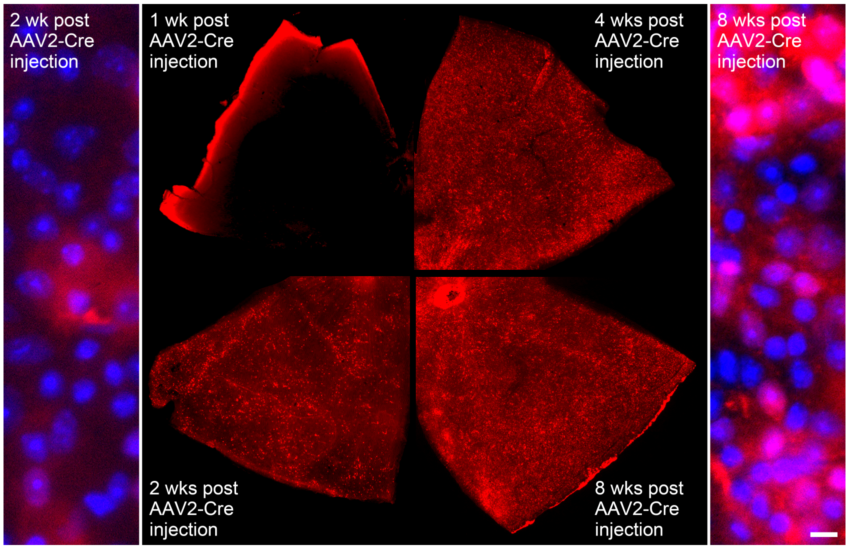

Supplemental Figure 1 Optimal viral transduction and gene expression following intravitreal injection of AAV2-Cre/GFP. Outer panels indicate high magnification images of cells from the ganglion cell layer of retinal whole mounts from *Rosa26-Tomato*$^{fl/fl}$ mice intravitreally injected with AAV2-Cre/GFP 2 and 8 weeks prior. (Scale bar: 10 μm) The inner panels illustrate low magnification images of whole-mounted retinas from the same *Rosa26-Tomato*$^{fl/fl}$ mice that were intravitreally injected 1, 2, 4, and 8 weeks prior to imaging. Expression of *td-Tomato* peaks as early as 4 weeks post AAV2-Cre injection.
